# Supplementary material for: The effectiveness of low-dosed outpatient biopsychosocial interventions compared to active physical interventions on pain and disability in adults with nonspecific chronic low back pain: A protocol for a systematic review with meta-analysis
Source: PLoS One. 2022 Sep 1;17(9):e0273983. doi: 10.1371/journal.pone.0273983 (PMC9436074; doi:10.1371/journal.pone.0273983)
Supplement: S1 Appendix — (DOCX) [file pone.0273983.s001.docx]

**Medline (Ovid):**

1. ("back disorder" or "back pain" or back-ache or backache or lbp).ti.
2. ("chronic back" or clbp or "chronic low back" or "chronic lower back").ti.
3. (dorsalgia or intervertebral or lumbago or "lumbal back" or lumbar or lumbo-sacral or lumbosacral or spinal or spine or spondylolisthesis).ti.
4. ("back pain" or "cauda equina" or "intervertebral disc" or "sciatic neuropathy" or "spinal diseases" or spine).sh.
5. 1 or 2 or 3 or 4
6. (exercis* or fitness or training).ab,ti.
7. (physical or physiotherapy).ab,ti.
8. (balance or proprioception or "sensorimotor training" or "stabili?ing exercis*").ab,ti.
9. (isometric or isotonic or "resistance training" or "weight lifting" or "muscle strength*").ab,ti.
10. (function* or "motor control" or "movement techniqu*" or neuromuscular).ab,ti.
11. ("tai chi" or "tai ji" or yoga).ab,ti.
12. ("abdominal exercis*" or core).ab,ti.
13. (adaptation or "aquatic exercis*" or calisthenics or circuit-based or plyometric).ab,ti.
14. (exercise or "physical fitness" or "physical therapy modalities" or recreation or yoga).sh.
15. 15 6 or 7 or 8 or 9 or 10 or 11 or 12 or 13 or 14
16. (ambulatory or outpatient or "primary care" or "primary health" or "primary healthcare" or "primary medical").ab,ti.
17. ("family medical practi*" or "general practi*" or gp or "physical therap*").ab,ti.
18. (interdisciplin* or multi-modal or multimodal or multi-professional or multicent$2 or multicomponent or multidisciplin* or multiprofessional).ab,ti.
19. (community or "social environment").ab,ti.
20. ("case management" or "directive counselling" or "long-term treatment" or "patient care").ab,ti
21. ("ambulatory care" or "patient care management").sh.
22. 16 or 17 or 18 or 19 or 20 or 21
23. "randomized controlled trial".sh.
24. (trial or random* or rct).ab,ti.
25. 23 or 24
26. (cbt or cognit* or behavio*).ab,ti.
27. ("bio psychosocial" or "biopsycho social" or biopsychosocial or "combined modality therapy" or "multidisciplinary rehabilitation" or psychosocial or "stratified primary care management").ab,ti.
28. (psycholog* or psychophysiology or psychotherapy or vocational).ab,ti.
29. (education or "health knowledge" or psychoeducation).ab,ti.
30. (meditation or "mind and body" or "mind-body" or relaxation or mindfulness).ab,ti.
31. ("acceptance and commitment therapy" or conditioning or "coping skill" or "functional recovery" or "functional rehabilitation" or "graded activity" or motivation or "recovery of function" or "self-care" or "self-efficacy" or "self-regulation training" or "social support").ab,ti.
32. ("cognitive behavioral therapy" or "patient education as topic" or "rehabilitation, vocational" or "social environment" or "social work").sh.
33. 26 or 27 or 28 or 29 or 30 or 31 or 32
34. 5 and 15 and 22 and 25 and 33
35. ("meta-analysis" or "observational study" or "scoping review" or review or "study protocol" or "systematic review" or "literature review").ti.
36. (acupuncture or ankylosing or Arthroplasty or blood or "dry cupping" or electrotherapy or fusion or "interspinous spacer" or "laser therapy" or "manipulative therapy" or "manipulative treatment" or "manual therapy" or massage or medication or microdiscectomy or oral or opioid* or "spinal cord compression" or "spinal fusion" or "spinal manipulation" or "spinal mobili*" or traction or stenosis or surgery or surgical).ti.
37. (adolescents or cancer or "cell therapy" or children or fractur* or hip or infection or neck or pain clinic or postoperative or pregnan* or spondylarthritis).ti.
38. 35 or 36 or 37
39. 34 not 38
40. **limit 39 to yr="1860 - 2021"**

**PubMed (NLM): up to 31December 2021**

**Date searched:**

Search: #40 #34 NOT #38 from 1000/1/1 - 2021/12/31

Search: #39 #34 NOT #38

Search: #38 #35 OR #36 OR #37

Search: #37 adolescents[ti] OR cancer[ti] OR "cell therapy"[ti] OR children[ti] OR fracture*[ti] OR hip[ti] OR infection[ti] OR neck[ti] OR "pain clinic"[ti] OR postoperative[ti] OR pregnan*[ti] OR spondylarthritis[ti]

Search: #36 acupuncture[ti] OR ankylosing[ti] OR Arthroplasty[ti] OR blood[ti] OR "dry cupping"[ti] OR electrotherapy[ti] OR fusion[ti] OR "interspinous spacer"[ti] OR "laser therapy"[ti] OR "manipulative therapy"[ti] OR "manipulative treatment"[ti] OR "manual therapy"[ti] OR massage[ti] OR medication[ti] OR microdiscectomy[ti] OR oral[ti] OR opioid*[ti] OR "spinal cord compression"[ti] OR "spinal fusion"[ti] OR "spinal manipulation"[ti] OR "spinal mobili*"[ti] OR traction [ti] OR stenosis[ti] OR surgery[ti] OR surgical[ti]

Search: #35 meta-analysis[ti] OR "observational study"[ti] OR "scoping review"[ti] OR review[ti] OR "study protocol"[ti] OR "systematic review"[ti] OR "literature review"[ti]

Search: #34 #5 AND #15 AND #22 AND #25 AND #33

Search: #33 #26 OR #27 OR #28 OR #29 OR #30 OR #31 OR #32

Search: #32 "cognitive behavioral therapy"[MeSH Terms] OR "patient education as topic"[MeSH Terms] OR "rehabilitation, vocational"[MeSH Terms] OR "social environment"[MeSH Terms] OR "social work"[MeSH Terms]

Search: #31 "acceptance and commitment therapy"[tiab] OR conditioning[tiab] OR "coping skill"[tiab] OR "functional recovery"[tiab] OR "functional rehabilitation"[tiab] OR "graded activity"[tiab] OR kinesiotherapy[tiab] OR motivation[tiab] OR "recovery of function"[tiab] OR "self-care"[tiab] OR "self-efficacy"[tiab] OR "self-regulation training"[tiab] OR "social support"[tiab]

Search: #30 meditation[tiab] OR "mind and body"[tiab] OR "mind-body”[tiab] OR relaxation[tiab] OR mindfulness[tiab]

Search: #29 education[tiab] OR "health knowledge"[tiab] OR psychoeducation[tiab]

Search: #28 "psycholog* "[tiab] OR psychophysiology[tiab] OR psychotherapy[tiab] OR vocational[tiab]

Search: #27 "bio psychosocial"[tiab] OR "biopsycho social"[tiab] OR biopsychosocial[tiab] OR "combined modality therapy"[tiab] OR "multidisciplinary rehabilitation"[tiab] OR psychosocial[tiab] OR "stratified primary care management"[tiab]

Search: #26 cbt[tiab] OR cognit*[tiab] OR behavio*[tiab]

Search: #25 #23 OR #24

Search: #24 "randomized controlled trials as topic"[MeSH Terms]

Search: #23 random*[tiab] OR rct[tiab] OR trial*[tiab]

Search: #22 #16 OR #17 OR #18 OR #19 OR #20 OR #21

Search: #21 "ambulatory care"[MeSH Terms] OR "patient care management"[MeSH Terms]

Search: #20 "case management"[tiab] OR "directive counselling"[tiab] OR "long-term treatment"[tiab] OR "patient care"[tiab]

Search: #19 community[tiab] OR "social environment"[tiab]

Search: #18 interdisciplin*[tiab] OR multi-modal[tiab] OR multimodal[tiab] OR multi-professional[tiab] OR multiprofessional[tiab] OR multicenter[tiab] OR multicentre[tiab] OR multicomponent[tiab] OR multidisciplin*[tiab]

Search: #17 "family medical practi*"[tiab] OR "general practi*"[tiab] OR gp[tiab] OR "physical therap*"[tiab]

Search: #16 ambulatory[tiab] OR outpatient[tiab] OR "primary care"[tiab] OR "primary health"[tiab] OR "primary healthcare"[tiab] OR "primary medical"[tiab]

Search: #15 #6 OR #7 OR #8 OR #9 OR #10 OR #11 OR #12 OR #13 OR #14

Search: #14 exercise [MeSH Terms] OR physical fitness [MeSH Terms] OR physical therapy modalities [MeSH Terms] OR recreation [MeSH Terms] OR yoga [MeSH Terms]

Search: #13 adaptation[tiab] OR "aquatic exercis*"[tiab] OR calisthenics[tiab] OR circuit-based[tiab] OR plyometric[tiab]

Search: #12 "abdominal exercis*"[tiab] OR core[tiab]

Search: #11 "tai chi"[tiab] OR "tai ji"[tiab] OR yoga[tiab]

Search: #10 function*[tiab] OR "motor control"[tiab] OR "movement techniqu*"[tiab] OR neuromuscular[tiab]

Search: #9 isometric[tiab] OR isotonic[tiab] OR "resistance training"[tiab] OR "weight lifting"[tiab] OR "muscle strength*"[tiab]

Search: #8 balance[tiab] OR proprioception[tiab] OR "sensorimotor training"[tiab] OR "stabilising exercis*"[tiab] OR "stabilizing exercis*"[tiab]

Search: #7 physical[tiab] OR physiotherapy[tiab]

Search: #6 exercis*[tiab] OR fitness[tiab] OR training[tiab]

Search: #5 #1 OR #2 OR #3 OR #4

Search: #4 "back pain"[MeSH Terms] OR "cauda equina"[MeSH Terms] OR "intervertebral disc"[MeSH Terms] OR "sciatic neuropathy"[MeSH Terms] OR "spinal diseases"[MeSH Terms] OR "spine"[MeSH Terms]

Search: #3 dorsalgia[ti] OR intervertebral[ti] OR lumbago[ti] OR "lumbal back"[ti] OR lumbar[ti] OR lumbo-sacral[ti] OR lumbosacral[ti] OR spinal[ti] OR spine[ti] OR spondylolisthesis[ti]

Search: #2 "chronic back"[ti] OR clbp[ti] OR "chronic low back"[ti] OR "chronic lower back” [ti]

Search: #1 "back disorder"[ti] OR "back pain"[ti] OR back-ache[ti] OR backache[ti] OR lbp[ti]

**Cochrane Central Register of Controlled Trials (CENTRAL) (Wiley).**

#1 ("back disorder" OR "back pain" OR back-ache OR backache OR lbp):ti

#2 ("chronic back" OR clbp OR "chronic low back" OR "chronic lower back"):ti

#3 (dorsalgia OR intervertebral OR lumbago OR "lumbal back" OR lumbar OR lumbo-sacral OR lumbosacral OR spinal OR spine OR spondylolisthesis):ti

#4 [mh "back pain"] OR [mh "cauda equina"] OR [mh "intervertebral disc"] OR [mh "sciatic neuropathy"] OR [mh "spinal diseases"] OR [mh spine]

#5 #1 OR #2 OR #3 OR #4

#6 (exercis* OR fitness OR training):ti,ab

#7 (physical OR physiotherapy):ti,ab

#8 (balance OR proprioception OR "sensorimotor training" OR "stabili?ing exercis*"):ti,ab

#9 (isometric OR isotonic OR "resistance training" OR "weight lifting" OR "muscle strength*"):ti,ab

#10 (function* OR "motor control" OR "motor control exercis*" OR "movement techniqu*" OR neuromuscular):ti,ab

#11 ("tai chi" OR "tai ji" OR yoga):ti,ab

#12 ("abdominal exercis*" OR core):ti,ab

#13 (adaptation OR "aquatic exercis*" OR calisthenics OR circuit-based OR plyometric):ti,ab

#14 [mh exercise] OR [mh "physical fitness"] OR [mh "physical therapy modalities"] OR [mh recreation] OR [mh yoga]

#15 #6 OR #7 OR #8 OR #9 OR #10 OR #11 OR #12 OR #13 OR #14

#16 (ambulatory OR outpatient OR "primary care" OR "primary health" OR "primary healthcare" OR "primary medical"):ti,ab

#17 ("family medical practi*" OR "general practi*" OR "general practitioner" OR gp OR "physical therap*"):ti,ab

#18 (interdisciplin* OR multi-modal OR multimodal OR multi-professional OR multicent$2 OR multicomponent OR multidisciplin* OR multiprofessional):ti,ab

#19 (community OR "social environment"):ti,ab

#20 ("case management" OR "directive counselling" OR "long-term treatment" OR "patient care"):ti,ab

#21 [mh "ambulatory care"] OR [mh "patient care management"]

#22 #16 OR #17 OR #18 OR #19 OR #20 OR #21

#23 (random* OR rct OR trial):ti,ab

#24 [mh "randomized controlled trials as topic"]

#25 #23 OR #24

#26 (cbt OR cognit* OR behavio*):ti,ab

#27 ("bio psychosocial" OR "biopsycho social" OR biopsychosocial OR "combined modality therapy" OR "multidisciplinary rehabilitation" OR psychosocial OR "stratified primary care management"):ti,ab

#28 (psycholog* OR psychophysiology OR psychotherapy OR vocational):ti,ab

#29 (education OR "health knowledge" OR psychoeducation):ti,ab

#30 (meditation OR "mind and body" OR "mind-body" OR relaxation OR mindfulness):ti,ab

#31 ("acceptance and commitment therapy" OR conditioning OR "coping skill" OR "functional recovery" OR "functional rehabilitation" OR "graded activity" OR motivation OR "recovery of function" OR "self-care" OR "self-efficacy" OR "self-regulation training" OR "social support"):ti,ab

#32 [mh "cognitive behavioral therapy"] OR [mh "patient education as topic"] OR [mh "rehabilitation, vocational"] OR [mh "social environment"] OR [mh "social work"]

#33 #26 OR #27 OR #28 OR #29 OR #30 OR #31 OR #32

#34 #5 AND #15 AND #22 AND #25 AND #33

#35 (meta-analysis OR "observational study" OR "scoping review" OR review OR "study protocol" OR "systematic review" OR "literature review"):ti

#36 (acupuncture OR ankylosing OR Arthroplasty OR blood OR "dry cupping" OR electrotherapy OR fusion OR "interspinous spacer" OR "laser therapy" OR "manipulative therapy" OR "manipulative treatment" OR "manual therapy" OR massage OR medication OR microdiscectomy OR oral OR opioid* OR "spinal cord compression" OR "spinal fusion" OR "spinal manipulation" OR "spinal mobili*" OR traction OR stenosis OR surgery OR surgical):ti

#37 (adolescents OR cancer OR "cell therapy" OR children OR fractur* OR hip OR infection OR neck OR "pain clinic" OR postoperative OR pregnan* OR spondylarthritis):ti

#38 #35 OR #36 OR #37

#39 #34 NOT #38

#40 #39 with Cochrane Library publication date to Dec 2021

**CINAHL – EBSCO**

S40 S39 Limiters - Published Date: -20211231

S39 S34 NOT S48

S38 S35 OR S36 OR S37

S37 TI adolescents OR TI cancer OR TI "cell therapy" OR TI children OR TI fractur* OR TI hip OR TI infection OR TI neck OR TI "pain clinic" OR TI postoperative OR TI pregnan* OR TI spondylarthritis

S36 TI acupuncture OR TI ankylosing OR TI Arthroplasty OR TI blood OR TI "dry cupping" OR TI electrotherapy OR TI fusion OR TI "interspinous spacer" OR TI "laser therapy" OR TI "manipulative therapy" OR TI "manipulative treatment" OR TI "manual therapy" OR TI massage OR TI medication OR TI microdiskectomy OR TI oral OR TI opioid* OR TI "spinal cord compression" OR TI "spinal fusion" OR TI "spinal manipulation" OR TI "spinal mobili*" OR TI traction OR TI stenosis OR TI surgery OR TI surgical

S35 TI "meta-analysis" OR TI "observational study" OR TI "scoping review" OR TI review OR TI "study protocol" OR TI "systematic review" OR TI "literature review"

S34 S5 AND S15 AND S22 AND S25 AND S33

S33 S26 OR S27 OR S28 OR S29 OR S30 OR S31 OR S32

S32 (MH "cognitive behavioral therapy"+) OR (MH "patient education as topic"+) OR (MH "rehabilitation, vocational"+) OR (MH "social environment"+) OR (MH "social support"+) OR (MH "social work"+)

S31 (TI "acceptance and commitment therapy" OR AB "acceptance and commitment therapy") OR (TI conditioning OR AB conditioning) OR (TI "coping skill" OR AB "coping skill") OR (TI "functional recovery" OR AB "functional recovery") OR (TI "functional rehabilitation" OR AB "functional rehabilitation") OR (TI "graded activity" OR AB "graded activity") OR (TI kinesiotherapy OR AB kinesiotherapy) OR (TI motivation OR AB motivation) OR (TI "recovery of function" OR AB "recovery of function") OR (TI "self-care" OR AB "self-care") OR (TI "self- efficacy" OR AB "self-efficacy") OR (TI "self-regulation training" OR AB "self-regulation training") OR (TI "social support" OR AB "social support")

S30 (TI "meditation" OR AB "meditation") OR (TI "mind and body" OR AB "mind and body") OR (TI "mind-body" OR AB "mind-body") OR (TI mindfulness OR AB mindfulness) OR (TI relaxation OR AB relaxation)

S29 (TI "education" OR AB "education") OR (TI "health knowledge" OR AB "health knowledge") OR (TI psychoeducation OR AB psychoeducation)

S28 (TI psycholog* OR AB psycholog* ) OR (TI psychophysiology OR AB psychophysiology) OR (TI psychotherapy OR AB psychotherapy) OR (TI vocational OR AB vocational)

S27 (TI "bio psychosocial" OR AB "bio psychosocial") OR (TI "biopsycho social" OR AB "biopsycho social") OR (TI biopsychosocial OR AB biopsychosocial) OR (TI "combined modality therapy" OR AB "combined modality therapy") OR (TI "multidisciplinary rehabilitation" OR AB "multidisciplinary rehabilitation") OR (TI psychosocial OR AB psychosocial) OR (TI "stratified primary care management" OR AB "stratified primary care management")

S26 (TI cbt OR AB cbt) OR (TI cognit* OR AB cognit*) OR (TI behavio* OR AB behavio* )

S25 S23 OR S24

S24 (MH "randomized controlled trials as topic"+)

S23 (TI random* OR AB random*) OR (TI rct OR AB rct) OR (TI trial OR AB trial)

S22 S16 OR S17 OR S18 OR S19 OR S20 OR S21

S21 (MH "ambulatory care"+) OR (MH "patient care management"+)

S20 (TI "case management" OR AB "case management") OR (TI "directive counselling" OR AB "directive counselling") OR (TI "long-term treatment" OR AB "long-term treatment") OR (TI "patient care" OR AB "patient care")

S19 (TI community OR AB community) OR ((TI "social environment" OR AB "social environment")

S18 (TI "interdisciplin*" OR AB "interdisciplin*") OR (TI multi-modal OR AB multi-modal) OR (TI multi-professional OR AB multi-professional) OR (TI multicenter OR AB multicenter) OR (TI multicentre OR AB multicentre) OR (TI multicomponent OR AB multicomponent) OR (TI "multidisciplin*" OR AB "multidisciplin*") OR (TI multimodal OR AB multimodal) OR (TI multiprofessional OR AB multiprofessional)

S17 (TI "family medical practi*" OR AB "family medical practi*") OR (TI "general practi*" OR AB "general practi*") OR (TI gp OR AB gp) OR (TI "physical therap*" OR AB "physical therap*")

S16 (TI ambulatory OR AB ambulatory) OR (TI outpatient OR AB outpatient) OR (TI "primary care" OR AB "primary care") OR (TI "primary health " OR AB "primary health") OR (TI "primary healthcare" OR AB "primary healthcare") OR (TI "primary medical " OR AB "primary medical")

S15 S6 OR S7 OR S8 OR S9 OR S10 OR S11 OR S12 OR S13 OR S14

S14 (MH exercise+) OR (MH "physical fitness"+) OR (MH "physical therapy modalities"+) OR (MH recreation+) OR (MH yoga+)

S13 (TI adaptation OR AB adaptation) OR (TI "aquatic exercis*" OR AB "aquatic exercis*") OR (TI calisthenics OR AB calisthenics) OR (TI circuit-based OR AB circuit-based) OR (TI plyometric OR AB plyometric)

S12 (TI "abdominal exercis*" OR AB "abdominal exercis*") OR (TI core OR AB core)

S11 (TI "tai chi" OR AB "tai chi") OR (TI "tai ji" OR AB "tai ji") OR (TI yoga OR AB yoga)

S10 (TI function* OR AB function*) OR (TI "motor control" OR AB "motor control") OR (TI "movement techniqu*" OR AB "movement techniqu*") OR (TI neuromuscular OR AB neuromuscular)

S9 (TI isometric OR AB isometric) OR (TI isotonic OR AB isotonic) OR (TI "muscle strength*" OR AB "muscle strength*") OR (TI "resistance training" OR AB "resistance training") OR (TI "weight lifting" OR AB "weight lifting")

S8 (TI balance OR AB balance) OR (TI proprioception OR AB proprioception) OR (TI "sensorimotor training" OR AB "sensorimotor training") OR (TI "stabili#ing exercis*" OR AB "stabili#ing exercis*")

S7 (TI physical OR AB physical) OR (TI physiotherapy OR AB physiotherapy)

S6 (TI exercis* OR AB exercis*) OR (TI fitness OR AB fitness) OR (TI training OR AB training)

S5 S1 OR S2 OR S3 OR S4

S4 (MH "back pain"+) OR (MH "cauda equina"+) OR (MH "intervertebral disc"+) OR (MH "sciatic neuropathy"+) OR (MH "spinal diseases"+) OR (MH spine+)

S3 TI dorsalgia OR TI intervertebral OR TI lumbago OR TI "lumbal back" OR TI lumbar OR TI lumbo-sacral* OR TI spinal OR TI spine OR TI spondylolisthesis

S2 TI "chronic back" OR TI clbp OR TI "chronic low back" OR TI "chronic lower back"

S1 TI "back disorder" OR TI "back pain" OR TI back-ache OR TI backache OR TI lbp

**PEDro (**[**http://www.pedro.org.au/**](http://www.pedro.org.au/)**): up to 31.12.2021**

**Date searched:**

Advanced Search

| **Search Fields** | **Search Terms** | **Results** |
| --- | --- | --- |
| Abstract & Title  AND  Body Part*  AND  Method | biopsychosocial  lumbar spine, sacro-iliac joint or pelvis  clinical trial |  |
| Abstract & Title  AND  Body Part*  AND  Method | psychosocial  lumbar spine, sacro-iliac joint or pelvis  clinical trial |  |

**Web of Science Core Collection (Clarivate)**

**Date searched: 19.01.22**

**41 #40 (Filter 2021)**

40 #39

39 #34 NOT #38

38 #35 OR #36 OR #37

37 TS=(adolescents OR cancer OR "cell therapy" OR children OR fractur* OR hip OR infection OR neck OR "pain clinic" OR postoperative OR pregnan* OR spondylarthritis )

36 TS=("acupuncture" OR ankylosing OR Arthroplasty OR blood OR "dry cupping" OR electrotherapy OR fusion OR "interspinous spacer" OR "laser therapy" OR "manipulative therapy" OR "manipulative treatment" OR "manual therapy" OR massage OR medication OR microdiskectomy OR oral OR opioid* OR "spinal cord compression" OR "spinal fusion" OR "spinal manipulation" OR "spinal mobili*" OR traction OR stenosis OR surgery OR surgical)

35 TS=("meta-analysis" OR "observational study" OR "scoping review" OR review OR "study protocol" OR "systematic review" OR "literature review" )

34 #5 AND #15 AND #22 AND #25 AND #33

33 #26 OR #27 OR #28 OR #29 OR #30 OR #31 OR #32

32 TS=("cognitive behavioral therapy" OR "patient education as topic" OR "rehabilitation, vocational" OR "social environment" OR "social work" )

31 TS=("acceptance and commitment therapy" OR conditioning OR "coping skill" OR "functional recovery" OR "functional rehabilitation" OR "graded activity" OR motivation OR "recovery of function" OR "self-care" OR "self-efficacy" OR "self-regulation training" OR "social support")

30 TS=(meditation OR "mind and body" OR "mind-body" OR relaxation OR mindfulness)

29 TS=(education OR "health knowledge" OR psychoeducation)

28 TS=(psycholog* OR psychophysiology OR psychotherapy OR vocational)

27 TS=("bio psychosocial" OR "biopsycho social" OR biopsychosocial OR "combined modality therapy" OR "multidisciplinary rehabilitation" OR psychosocial OR "stratified primary care management")

26 TS=(cbt OR cognit* OR behavio*)

25 #23 OR #24

24 TS=("randomized controlled trials as topic" )

23 TS=( random* OR rct OR trial)

22 #16 OR #17 OR #18 OR #19 OR #20 OR #21

21 TS=("ambulatory care" OR ""patient care management")

20 TS=("case management" OR "directive counselling" OR "long-term treatment" OR "patient care")

19 TS=(community OR "social environment" )

18 TS=(interdisciplin* OR multi-modal OR multimodal OR multi-professional OR multicentre OR multicenter OR multicomponent OR multidisciplin* OR multiprofessional)

17 TS=("family medical practi*" OR "general practi*" OR gp OR "physical therap*")

16 TS=( ambulatory OR outpatient OR "primary care" OR "primary health " OR "primary healthcare" OR "primary medical")

15 #6 OR #7 OR #8 OR #9 OR #10 OR #11 OR #12 OR #13 OR #14

14 TS=(exercise OR "physical fitness" OR "physical therapy modalities" OR recreation OR yoga)

13 TS=(adaptation OR "aquatic exercis*" OR calisthenics OR circuit-based OR plyometric)

12 TS=("abdominal exercis*" OR core)

11 TS=("tai chi" OR "tai ji" OR yoga)

10 TS=(function* OR "motor control" OR "motor control exercis*" OR "movement techniqu*" OR neuromuscular )

9 TS=(isometric OR isotonic OR "resistance training" OR "weight lifting" OR "muscle strength*")

8 TS=(balance OR proprioception OR "sensorimotor training" OR "stabili?ing exercis*")

7 TS=(physical OR physiotherapy )

6 TS=(exercis* OR fitness OR training)

5 #1 OR #2 OR #3 OR #4

4 TS=("back pain" OR "cauda equina" OR "intervertebral disc" OR "sciatic neuropathy" OR "spinal diseases" OR spine)

3 TI=(dorsalgia OR intervertebral OR lumbago OR "lumbal back" OR lumbar OR lumbo-sacral OR lumbosacral OR spinal OR spine OR spondylolisthesis)

2 TI=("chronic back" OR clbp OR "chronic low back" OR "chronic lower back")

1 TI=("back disorder" OR "back pain" OR back-ache OR backache OR lbp)
